# Supplementary material for: Potent Activation of Human but Not Mouse TRPA1 by JT010
Source: Int J Mol Sci. 2022 Nov 18;23(22):14297. doi: 10.3390/ijms232214297 (PMC9695908; doi:10.3390/ijms232214297)
Supplement: Supplementary file 1 [file ijms-23-14297-s001.zip › ijms-2007212-supplementary.pdf]

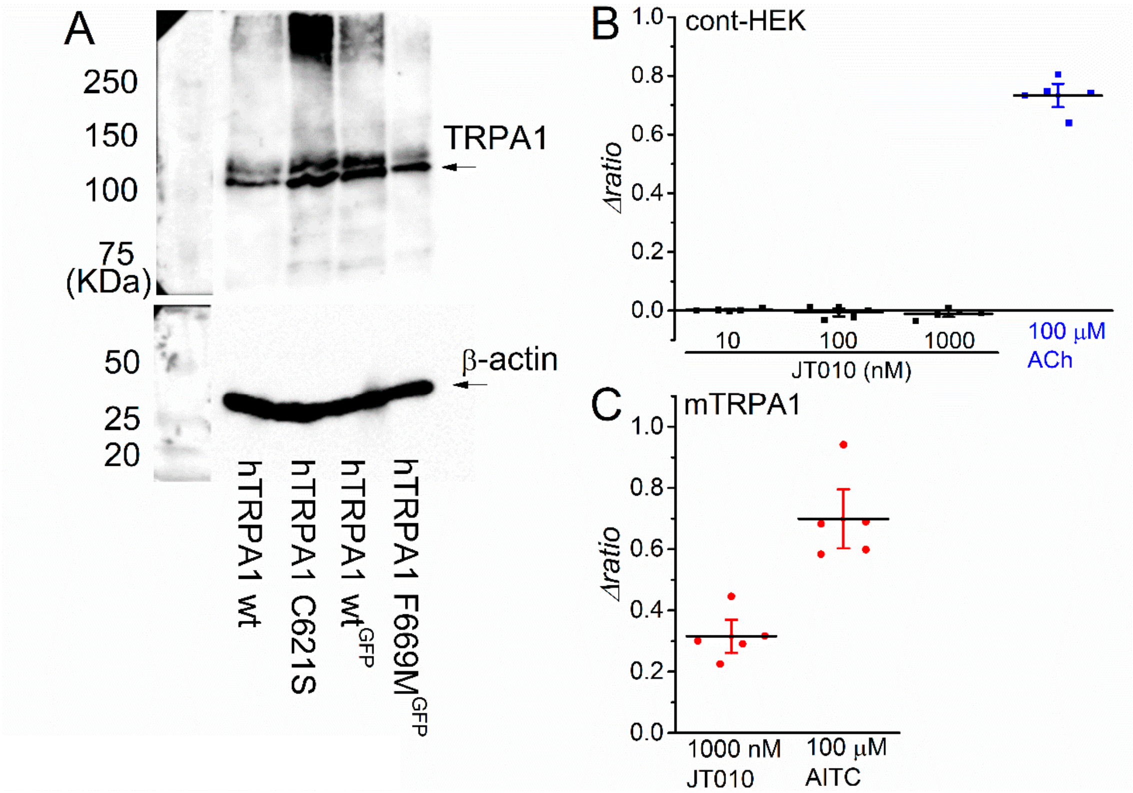

Supplementary Figure S1. (A) Expression of wild and mutant hTRPA1. Wild (wt) and mutant hTRPA1 (C621S) proteins for Ca^{2+} measurement and wild (wt^{GFP}) and mutant hTRPA1 ($F669M^{GFP}$) for patch-clamp experiments were assayed by western blotting (WB) with an antibody against C-terminal hTRPA1. As a control, β-actin protein level was also detected. A representative WB data set is shown out of three independent experiments (See Methods for details). (B) No effects of JT010 on native HEK (cont-HEK) cells. JT010 at a concentration ranging from 10 to 1000 nM was applied, and Ca^{2+} response was monitored. The peak JT010-induced Ca^{2+} response (Δratio) in cont-HEK cells (five independent experiments) is summarized. At the end of each experiment, 100 μM acetylcholine (ACh) was applied to confirm the Ca^{2+} response. (C) Effects of a high JT010 concentration on HEK-mTRPA1 cells. JT010 at 1000 nM was applied, and Ca^{2+} response was monitored. The peak JT010-induced Ca^{2+} response (Δratio) in HEK-mTRPA1 cells (five independent experiments) is summarized. At the end of the experiment, 100 μM AITC was applied to confirm mTRPA1 expression, and the expression is summarized.

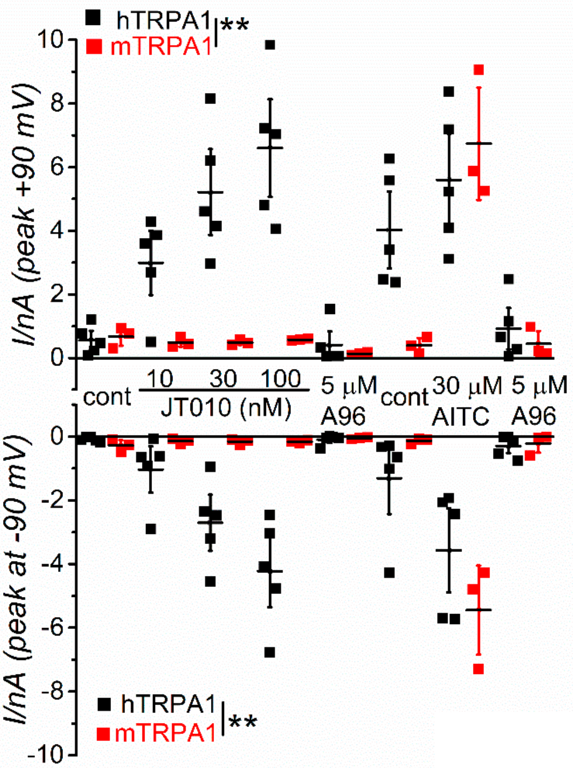

Supplementary Figure S2. Effects of JT010 on hTRPA1 and mTRPA1 in the absence of intracellular Ca^{2+}. HEK cells expressing hTRPA1 and mTRPA1 were superfused with SBS without Ca^{2+}. The pipette solution contained 1 mM EGTA to chelate free Ca^{2+} in the solution. Ramp waveform voltage pulses from −110 to +90 mV for 300 ms were applied every 5 s at a holding potential of -10 mV. At the end of each experiment, AITC and A96 were applied to test the expression of hTRPA1 and mTRPA1. The peak JT010- and AITC-induced currents are summarized (five and three independent experiments for hTRPA1 and mTRPA1, respectively). Two-way ANOVA (+90 mV): **p<0.0001, F=32.5 (species); **p<0.0001, F=14.9 (treatments); **p<0.0001, F=6.36 (interaction). Two-way ANOVA (-90 mV): **p<0.004, F=9.14 (species); **p<0.0001, F=14.6 (treatments); **p=0.00015, F=5.33 (interaction). Vertical bars = SEM.

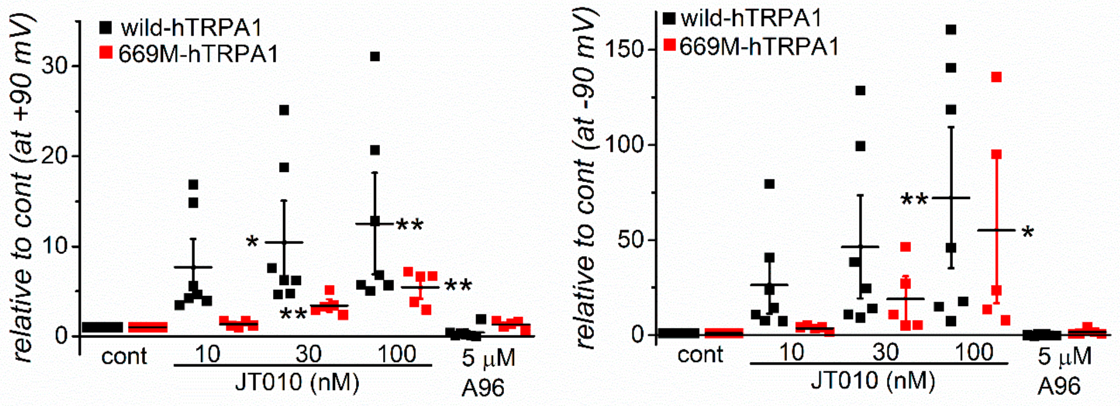

Supplementary Figure S3. Each current amplitude of wild and mutant hTRPA1 shown in Figure 2C and Figure 5C was normalized with that in control and is summarized as the relative amplitude change under each treatment. Dunnett's multiple comparison test was applied to each TRPA1. *p=0.0290 and **p=0.00637 for 30 and 100 nM JT010, respectively in wild-hTRPA1 (+90 mV). **p=0.00396 and **p<0.0001 for 30 and 100 nM JT010, respectively in 669M-hTRPA1 (+90 mV). **p=0.00552 for 100 nM JT010 in wild-hTRPA1 (-90 mV). *P=*0.0156* for 100 nM JT010 in 669M-hTRPA1 (-90 mV). Vertical bars = SEM.
